# Cobblestone-like Gastric Mucosal Changes on Endoscopy in Dogs with a History of Prolonged Proton Pump Inhibitor Therapy

**DOI:** 10.3390/ani16030406

**Published:** 2026-01-28

**Authors:** Martine Dominique Didier, Laura Zagnoli, Deborah Cattaneo, Silvia Lucia Benali, Enrico Bottero

**Affiliations:** 1 Clinica Veterinaria Gran Sasso, 20131 Milan, Italy; 2 Endovet Professional Association, 00149 Rome, Italy; laurazagnoli85@gmail.com (L.Z.); debo_cat@yahoo.it (D.C.); botvet@libero.it (E.B.); 3 MYLAV Veterinary Diagnostic Laboratory, 20017 Milan, Italy; silviabenali@mylav.net

**Keywords:** dog, endoscopy, cobblestone-like gastric mucosa, hypertrophic gastropathy, proton pump inhibitor, omeprazole, retrospective study

## Abstract

This study describes the clinicopathological features of seven canine cases showing a diffuse cobblestone-like gastric mucosal pattern on endoscopy. Cases were retrospectively retrieved from endoscopic databases (2017–2025). Clinical data, treatment history, endoscopic findings, and histology were reviewed. Endoscopically, all dogs exhibited thickened, irregular, and poorly distensible gastric folds. Histopathologic examination showed mild-to-moderate foveolar hyperplasia, variable cystic dilation of the fundic glands, mild chronic lymphoplasmacytic inflammation, and interstitial fibrosis. Parietal-cell population was variably increased and predominant (hyperplasia). Because these features can overlap widely among reactive and hyperplastic gastropathies, interpretation required correlation with clinical and endoscopic findings in addition to histopathology. All dogs had a history of prolonged omeprazole administration, and most showed clinical improvement after dose reduction or treatment withdrawal. Follow-up endoscopy in two dogs documented divergent outcomes, with marked improvement in one dog and only minimal changes in the other. These findings suggest that this cobblestone-like pattern represents a benign, reactive, and potentially regressive gastropathy, possibly associated with chronic acid suppression. Recognition of this appearance may assist clinicians in differentiating reactive gastropathy from proliferative or neoplastic conditions and supports prudent use of long-term proton pump inhibitors in dogs with chronic gastrointestinal disease.

## Simple Summary

Gastric mucosal hypertrophy in dogs is an uncommon condition that may resemble more serious disorders such as gastric neoplasia. This study describes seven canine cases characterized by a cobblestone-like thickening of the gastric mucosa observed during endoscopy. The aim of our study was to better characterize the clinical, endoscopic, and histopathologic features of this pattern and to investigate its potential relationship with prolonged proton pump inhibitor (PPI) therapy. Histological examination revealed superficial and foveolar hyperplasia, with occasional cystic dilation of fundic glands and hyperplasia of parietal cells, and no evidence of neoplasia. Most dogs improved clinically after omeprazole withdrawal or tapering. Follow-up endoscopy, available in two dogs, showed marked improvement in the dog re-evaluated after six months, whereas only minimal changes were observed in the dog re-evaluated after three months. These findings suggest that this pattern represents a benign, potentially regressive reactive gastropathy possibly related to chronic acid suppression. Recognizing this appearance may alert veterinarians to consider reactive causes in the differential diagnosis; however, endoscopic findings alone are insufficient to reliably distinguish reactive from proliferative or neoplastic gastric disease, and histopathologic evaluation of gastric biopsy samples is required for definitive diagnosis.

## 1. Introduction

Endoscopic evaluation of the gastric mucosa in dogs frequently reveals nonspecific abnormalities, and histopathologic assessment is often required to establish a definitive diagnosis. The most common endoscopic findings in inflammatory gastropathies include mucosal edema, hyperemia, erosions, discoloration, and cerebriform patterns, none of which are specific to a particular disease [1]. In contrast, diffuse thickening of the gastric folds with a cobblestone-like appearance is considered an uncommon finding in dogs and has been reported both in benign gastric disorders and in infiltrative neoplastic conditions such as lymphoma and carcinoma [2,3]. Non-neoplastic diseases presenting with a similar mucosal thickening, such as reactive hypertrophic or Ménétrier-like gastropathies, are only sporadically described and remain poorly characterized with respect to their clinicopathologic features and outcome [1,3,4]. More recently, a single canine case report described fundic gland polyps associated with long-term proton pump inhibitor administration, with clinical improvement after drug discontinuation, suggesting a possible association between chronic acid suppression and reactive gastric mucosal changes in dogs [5]. The limited number of veterinary reports, combined with the potential overlap in endoscopic appearance between benign reactive changes and infiltrative disorders, often complicates diagnostic interpretation. In human medicine, comparable endoscopic patterns have been documented in chronic inflammatory gastropathies and in drug-induced conditions, including those associated with prolonged proton pump inhibitor (PPI) therapy. In human medicine, reported histologic alterations include foveolar hyperplasia, parietal cell hypertrophy with cytoplasmic protrusion into the glandular lumen, and cystic dilation of the fundic glands, reflecting altered glandular architecture secondary to chronic acid suppression [6,7,8,9]. In dogs, several hypertrophic or hyperplastic gastropathies, including Ménétrier-like disease and chronic hypertrophic pyloric gastropathy, may exhibit overlapping endoscopic and histologic features with reactive or PPI-associated changes, making differentiation among these entities challenging [1,10]. Because long-term PPI use in humans is known to induce a spectrum of reactive histologic changes, including foveolar hyperplasia and glandular dilation [6,7,8,9], similar alterations may theoretically occur in predisposed veterinary patients undergoing prolonged acid-suppressive therapy. The present retrospective study describes the clinical, endoscopic, and histopathologic findings in a group of dogs exhibiting a cobblestone-like gastric mucosal appearance and investigates whether these lesions may represent a reactive gastropathy potentially associated with prolonged PPI administration.

## 2. Materials and Methods

This retrospective multicenter study included client-owned dogs that underwent diagnostic gastroscopy at the internal medicine services of three referral hospitals in Italy between 2017 and 2025. Gastroscopies were performed using flexible videoendoscopes available at each center; although equipment brands and endoscope diameters varied according to availability and patient size, standard diagnostic protocols were applied across centers. Endoscopic databases were searched using the keywords “cobblestone-like appearance,” “gastropathy,” and “cerebriform pattern.” At each participating center, the database search was performed by the local endoscopist responsible for clinical record archiving. Terminology was not prospectively standardized across centers, and case identification relied on the keywords used in routine clinical reports. Only gastroscopy reports and associated image archives labelled with the selected keywords were screened; a systematic review of all gastroscopic procedures performed during the study period was not undertaken. Therefore, cases not described using these terms may have been missed. All procedures were performed for diagnostic purposes; therefore, formal ethical approval was not required in accordance with national regulations. Written informed owner consent was obtained at the time of gastroscopy. Dogs were included if the gastric mucosa exhibited markedly thickened, non-distensible folds after insufflation, with an irregular or corrugated (“cobblestone-like”) surface involving the majority of the gastric body (operationally defined as approximately ≥75% of the gastric body), thereby distinguishing a diffuse mucosal pattern from focal or segmental lesions. Dogs with endoscopic alterations suggestive of focal masses, polypoid lesions, parasitic infestations, ulcers, or foreign bodies, as well as those with a histological diagnosis of gastric neoplasia or infectious/parasitic disease, were excluded. Only cases in which at least six biopsy samples had been collected from multiple gastric regions were included to ensure adequate histologic assessment. Biopsy samples were obtained from the fundus, body, and antral/pyloric regions in all dogs. All samples were routinely oriented on cellulose acetate paper prior to fixation to optimize histologic assessment. Cases were excluded if a diffuse cobblestone-like mucosal pattern was absent, if focal or mass-like lesions were identified, or if histologic examination revealed neoplasia, infectious or parasitic disease. Only cases fulfilling both the endoscopic and histologic inclusion criteria were included in the final analysis. For each included dog, available clinical data were collected, including signalment, reason for endoscopy, predominant clinical signs and duration, and results of diagnostic tests (complete blood count, serum biochemistry, and abdominal ultrasonography when available). Details regarding the indication for omeprazole therapy, including empiric treatment for chronic vomiting or suspected gastric ulceration, were also recorded on a per-case basis. Details of ongoing or previous medical treatments and follow-up were recorded. Follow-up information was obtained by telephone interviews with the owners of the selected cases. Data regarding ongoing or prior proton pump inhibitor (PPI) therapy were recorded for all dogs, including treatment duration (in days), dosing frequency (once daily vs twice daily), and information on concurrent medications; these data were available for all cases and are reported accordingly. Information regarding prior courses of proton pump inhibitors, previous use of potassium-competitive acid blockers (PCABs), or H2-receptor antagonists was not consistently documented and could not be reliably retrieved for all cases. Clinical evolution after treatment discontinuation and the availability and results of any follow-up endoscopic examinations were also recorded. Endoscopic recordings and images were retrospectively reviewed by two experienced operators to confirm inclusion criteria and describe the extent and distribution of mucosal changes. Archived gastric biopsy slides were re-evaluated by a single board-certified veterinary pathologist (Dipl. ECVP). Histopathologic evaluation and diagnosis of gastrointestinal inflammation were performed according to the World Small Animal Veterinary Association (WSAVA) criteria [11]. In addition, gastric mucosal changes previously described in association with prolonged PPI therapy were specifically assessed, including foveolar epithelial hyperplasia, parietal-cell hyperplasia, and cystic dilatation of fundic glands. Clinical, endoscopic, and histopathological data were entered into a dedicated spreadsheet (Microsoft Excel^®^) and analyzed using descriptive statistics. Continuous variables are reported as mean ± standard deviation (SD) or median (range), whereas categorical variables are expressed as absolute numbers and percentages.

## 3. Results

**Figure 1:**
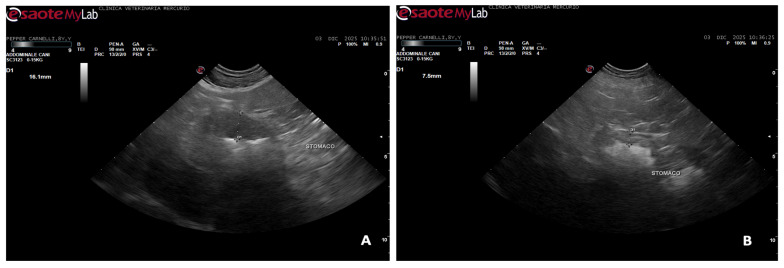
(**A**,**B**) Abdominal ultrasonography revealed marked thickening of the gastric wall, predominantly involving the mucosal layer of the fundus (**A**) and mild thickening of the mucosal layer of the gastric body (**B**), with loss of normal wall layering. No focal masses or evidence of transmural infiltration were detected.

A total of 4520 canine gastroscopies were performed during the study period. Database screening using predefined keywords identified seven dogs (0.15%) that met the inclusion criteria and were included in the analysis. The population comprised five males and two females, with a median age of 9 years (range 5–13). Breeds represented were Labrador Retriever (n = 1), Boxer (n = 1), Toy Poodle (n = 1), Yorkshire Terrier (n = 2), Chihuahua (n = 1), and Vizsla (n = 1). Body weight ranged from 2.5 to 33 kg (median 6 kg). All dogs presented with chronic vomiting, which was occasionally associated with diarrhea (2/7) (Table 1). Physical examination was unremarkable in most cases, although mild abdominal discomfort and dehydration were noted in two dogs. Abdominal ultrasonography revealed gastric wall thickening in all dogs, more evident in the body and fundic regions. Preservation of normal wall layering was observed in most cases, whereas segmental loss of layering was detected in two dogs, with no evidence of focal masses or transmural infiltration (Figure 1).

Other abdominal findings were nonspecific (e.g., a small endoluminal gallbladder wall lesion and variable urinary bladder distension), and laboratory analyses were within normal limits for all subjects. At the time of endoscopy, all dogs were receiving omeprazole at a dosage of approximately 1 mg/kg, administered once daily in five dogs and twice daily in two dogs. The indication for omeprazole therapy was available for all dogs and is summarized in Table 1. The duration of proton pump inhibitor (PPI) therapy ranged from 100 to 730 days. In most cases, omeprazole was initiated empirically for chronic vomiting, while one dog received therapy for suspected gastric ulceration and one for vomiting refractory to dietary and antiemetic management (Table 1). Concurrent medications were administered in four dogs and included antibiotics, steroids, cyclosporine, or tylosin.

**Figure 2:**
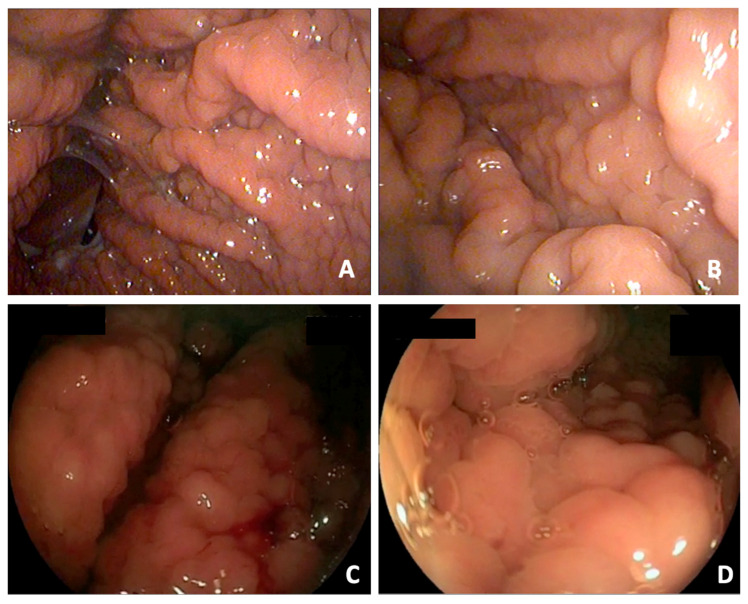
(**A**–**D**) Endoscopic views of the gastric body showing a markedly thickened, cobblestone-like mucosal surface with enlarged, poorly distensible folds. The mucosa appears edematous and moderately hyperemic, with occasional mild friability on contact.

A cobblestone-like surface pattern was evident in the gastric body of all dogs; in two cases, the alterations extended to the fundus, while the pyloric area appeared normal in all dogs. Lesions did not involve the cardia or incisura in any dog. Despite a macroscopically normal appearance, the antral and pyloric regions were routinely sampled as part of the standardized endoscopic protocol. The gastric folds were enlarged and poorly distensible, and the mucosa exhibited moderate to marked edema and hyperemia, occasionally with mild friability upon contact (Figure 2).

**Figure 3:**
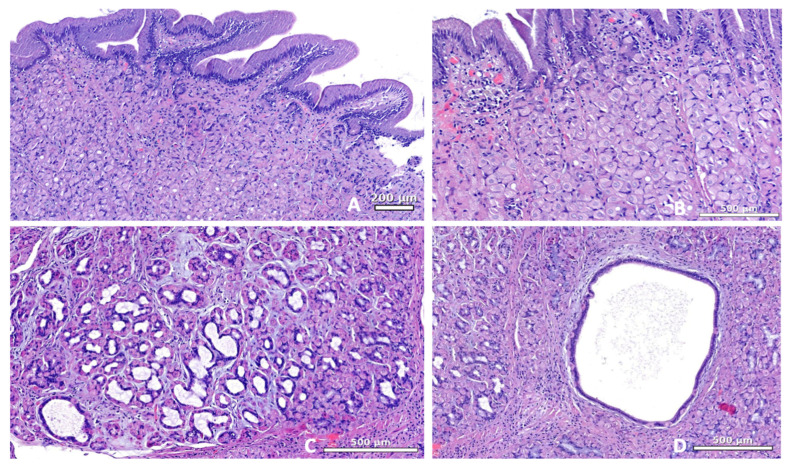
Gastric mucosa. (**A**) Hyperplasia of the superficial epithelium with formation of papillary projections (Hematoxylin-Eosin stain, 80×). (**B**) Gastric pits are lined by a predominance of parietal cells (hyperplasia). There is mild superficial lymphoplasmacytic inflammation (Hematoxylin–Eosin stain, 100×). Variable degree of dilation of fundic glands (**C**) with cystic formation (**D**) (Hematoxylin–Eosin stain, 100×).

No erosions, ulcers, or mass lesions were identified. All biopsy samples (n = 7) were considered adequate for diagnostic evaluation. Histopathological examination revealed mild foveolar hyperplasia in all dogs, occasionally forming microscopic, small polypoid projections (Table 2), and cystic gland dilation in five cases; this finding was absent in cases 1 and 7 (Figure 3).

The parietal-cell hyperplasia was mild to moderate in 6 cases. A mild-to-moderate lymphoplasmacytic infiltrate was present in the lamina propria of all dogs, variably admixed with scattered neutrophils, and accompanied by mild interstitial fibrosis. No mucosal atrophy, ulceration, or neoplastic changes were identified.

Clinical follow-up information was available for all dogs, with a follow-up duration ranging from 1 to 8 months (median 4 months). Improvement of clinical signs was reported in most dogs (5/7), whereas partial clinical improvement was observed in two dogs (2/7). Two dogs (cases 6 and 7) underwent follow-up endoscopy six and three months after discontinuation of omeprazole, respectively. In case 7, only minimal endoscopic improvement was observed, whereas in case 6, the gastric mucosa appeared markedly improved and almost normal. Histologic examination of the follow-up samples revealed findings comparable to those of the initial biopsies, with persistent epithelial and glandular alterations. No relapses or progressive changes were observed during the follow-up period.

## 4. Discussion

In human medicine, hypertrophic and hyperplastic gastropathies comprise a heterogeneous group of disorders ranging from reactive foveolar hyperplasia to preneoplastic or neoplastic proliferations, such as those described in cases of Ménétrier’s disease [4]. Although these entities may share similar endoscopic features, their underlying mechanisms differ and are often related to chronic inflammation, altered epithelial regeneration, or trophic effects secondary to prolonged acid suppression [7,8]. In dogs, comparable hypertrophic-like gastric changes have been reported only sporadically, often inconsistently, and with limited correlation between endoscopic and histologic findings [1,2,3]. In our case series, histopathological evaluation commonly revealed parietal cell hyperplasia and cystic dilation of the fundic glands, together with mild superficial and foveolar epithelial changes that are nonspecific and may overlap with other forms of chronic reactive gastropathy. Although these alterations have been reported as characteristic, they may be inconsistently present in hyperplastic gastropathies of different etiologies and are not pathognomonic for PPI-associated gastropathy; therefore, clinicopathologic correlation is required for appropriate interpretation. The preserved mucosal architecture and absence of dysplasia or neoplasia further support a chronic, reactive, non-proliferative condition rather than a neoplastic disorder. Endoscopically, the presence of thickened, irregular folds with a cobblestone-like appearance represented an uncommon yet consistent pattern. Similar findings have been associated in previous veterinary reports with Ménétrier-like disease or infiltrative neoplasia [2,3], both of which were excluded in our cases based on histology. Comparable cobblestone-like mucosal changes have also been described in humans receiving long-term PPI therapy [7,12]. In dogs, similar gastric alterations have been reported only sporadically and mainly in association with profound acid suppression, including cases treated with potassium-competitive acid blockers, suggesting that the underlying mechanism may be related to acid suppression rather than to a specific drug class [5,9]. However, these changes are nonspecific for PPI-associated mucosal injury and cannot be used as standalone indicators of drug-associated gastropathy. All dogs had received omeprazole for at least one month prior to endoscopy. Importantly, in most dogs, omeprazole has been initiated empirically for chronic vomiting rather than for a confirmed acid-related disorder (Table 1), supporting the possibility that PPI therapy may reflect underlying disease severity or chronicity rather than being the primary cause of the observed mucosal changes. Given that chronic inflammatory gastropathy is far more common in dogs than drug-induced reactive gastropathy, the endoscopic changes observed here may reflect one manifestation of an underlying chronic inflammatory process rather than a direct consequence of PPI therapy. Accordingly, these macroscopic features cannot be considered pathognomonic for PPI-associated mucosal changes. Although omeprazole is widely used in small-animal practice, lesions resembling those described in this study appear to be rare among dogs undergoing endoscopy. In human medicine, susceptibility to PPI-associated gastric alterations is believed to depend largely on individual predisposition rather than on dosage or treatment duration [8], and the rarity of this endoscopic pattern in our population supports a similar interpretation. A limitation of this study is the absence of precise data on the total number of dogs exposed to long-term PPI therapy during the study period evaluated. For this reason, the analysis focused primarily on the macroscopic gastric alterations, which are infrequently reported. Additionally, we could not assess the potential association between omeprazole exposure and gastric polyps; although such a link has been described in a canine case report [5], no dog in our series exhibited endoscopically visible polyps. Most dogs showed partial or complete clinical improvement after PPI withdrawal; however, the retrospective nature of the study precludes establishing causality. Precise temporal relationships between PPI initiation and endoscopic evaluation, as well as between PPI withdrawal and clinical response, could not be consistently established due to incomplete timing data in the medical records, further limiting causal inference. Dietary adjustments, concurrent treatments, or restoration of microbial balance may have contributed to symptom resolution; however, dietary history and direct assessment of microbial changes were not available in the present study. This is particularly relevant given the dysbiotic effects described in dogs undergoing prolonged omeprazole therapy [13,14,15]. Finally, although initial histopathologic evaluations were performed without knowledge of PPI exposure, the retrospective review of biopsy samples was conducted with access to clinical information, which may have introduced interpretative bias. Accordingly, given the retrospective study design and the presence of potential confounding factors, the findings should be interpreted as hypothesis-generating rather than as evidence of a causal relationship between proton pump inhibitor administration and gastric mucosal changes. The observed discordance between endoscopic improvement and persistent histological abnormalities may reflect sampling variability, delayed mucosal remodeling, or ongoing low-grade inflammation; therefore, the present results support the possibility of partial regression in some dogs rather than consistent or complete reversibility. Follow-up endoscopic evaluation was available in only two dogs. In case 6, follow-up endoscopy was performed six months after omeprazole discontinuation, whereas in case 7, it was performed three months after drug withdrawal. Clinical improvement was reported in both dogs, although the timing and extent of symptom resolution varied and were influenced by concurrent management changes. Histologic reassessment revealed findings comparable to the initial biopsies, consistent with the gradual and often delayed histologic normalization described in reports of PPI-associated gastropathy [6,15,16]. These observations suggest that mucosal remodeling following chronic acid-suppressive therapy may progress slowly or remain clinically silent for extended periods. Several histologic features in our cases overlapped with those described in human PPI-associated gastropathy, and a similar association has been reported in a dog developing multiple endoscopically visible fundic gland polyps after prolonged omeprazole therapy [5]. Taken together, despite the limitations inherent to the retrospective design and variability in clinical information, the consistent combination of endoscopic and histologic findings across dogs supports the interpretation that these lesions likely represent a reactive, rare, and potentially regressive mucosal response to prolonged acid suppression or chronic irritative/pharmacologic stimuli related to long-term acid-suppressive therapy, rather than a distinct pathological entity.

## 5. Conclusions

This series highlights a distinctive cobblestone-like endoscopic appearance in dogs, associated with mild, predominantly reactive histologic alterations that required clinical and endoscopic correlation for accurate interpretation. All dogs were undergoing long-term omeprazole therapy at the time of diagnosis, and the partial improvement observed after drug withdrawal supports a possible association with chronic acid suppression. These findings underline the importance of prudent long-term use of PPIs and emphasize the need for prospective, standardized studies to determine the underlying mechanisms and the clinical relevance of this rare endoscopic presentation.

## Figures and Tables

**Table 1:** Clinical data of dogs with cobblestone-like gastric mucosa.

Case	Breed/Sex/Age (y)	Weight (kg)	Main Signs	Indication for PPI Therapy	Duration of PPI Therapy (Days) and Dose	Concurrent Medications	Ultrasonographic Gastric Wall Findings	Follow-Up Outcome
1	Labrador M, 8	33	Vomiting	Suspected gastric ulceration	730 days (1 mg/kg BID)	No	Gastric mucosal thickening with loss of wall layering	Partial clinical improvement after treatment discontinuation
2	Boxer M, 11	30	Vomiting, diarrhea	Empiric treatment for chronic vomiting	150 days (1 mg/kg BID)	Antibiotics	Gastric mucosal thickening	Improvement of clinical signs after treatment discontinuation
3	Toy Poodle F, 9	6	Vomiting	Empiric treatment for chronic vomiting	730 days (1 mg/kg SID)	Cyclosporine	Gastric mucosal thickening	Partial clinical improvement after treatment discontinuation
4	Yorkshire Terrier M, 12	4	Vomiting	Vomiting refractory to dietary modification and antiemetic therapy	100 days (1 mg/kg SID)	No	Gastric mucosal thickening	Improvement of clinical signs after treatment discontinuation
5	Yorkshire Terrier M, 13	3.8	Vomiting, diarrhea	Empiric treatment for chronic vomiting	150 days (1 mg/kg SID)	Steroids, antibiotics	Gastric mucosal thickening	Improvement of clinical signs after treatment discontinuation
6	Chihuahua F, 7	2.5	Vomiting	Empiric treatment for chronic vomiting	100 days (1 mg/kg SID)	No	Gastric mucosal thickening	Improvement of clinical signs after treatment discontinuation
7	Vizsla M, 5	25	Vomiting	Empiric treatment for chronic vomiting	150 days (1 mg/kg SID)	Tylosin	Gastric mucosal thickening with loss of wall layering	Improvement of clinical signs after treatment discontinuation

**Table 2:** Histopathological findings of gastric biopsies.

Case	Superficial and Foveolar Hyperplasia	Cystic Dilatation of Fundic Glands	Parietal Cell Hyperplasia	Inflammatory Infiltrate (Type/Grade)	Fibrosis
1	Mild	Absent	Mild	Lymphoplasmacytic, mild	Mild
2	Mild	Present	Moderate	Lymphoplasmacytic ± neutrophilic, mild–moderate	Mild
3	Mild	Present	Moderate	Lymphoplasmacytic, mild–moderate	Moderate
4	Mild	Present	Moderate	Plasmacytic, mild–moderate	Mild
5	Mild	Present	Mild	Lymphoplasmacytic, mild–moderate	Mild–moderate
6	Mild	Present	Mild	Lymphoplasmacytic, mild–moderate	Mild
7	Mild	Absent	Mild	Lymphoplasmacytic, mild	Mild

## Data Availability

The data presented in this study are available on request from the corresponding author. The data are not publicly available due to privacy restrictions.

## Abbreviation

The following abbreviation is used in this manuscript:

PPI	Proton pump inhibitor
